# Light‐Based 3D Multi‐Material Printing of Micro‐Structured Bio‐Shaped, Conducting and Dry Adhesive Electrodes for Bioelectronics

**DOI:** 10.1002/advs.202306424

**Published:** 2024-01-22

**Authors:** Antonio Dominguez‐Alfaro, Eleni Mitoudi‐Vagourdi, Ivan Dimov, Matias L. Picchio, Naroa Lopez‐Larrea, Jon Lopez de Lacalle, Xudong Tao, Ruben Ruiz‐Mateos Serrano, Antonela Gallastegui, Nikolaos Vassardanis, David Mecerreyes, George G. Malliaras

**Affiliations:** ^1^ Electrical Engineering Division Department of Engineering University of Cambridge 9 JJ Thomson Ave Cambridge CB3 0FA UK; ^2^ POLYMAT University of the Basque Country UPV/EHU Avenida Tolosa 72 Donostia‐San Sebastián Gipuzkoa 20018 Spain; ^3^ VASSARDANIS L.P. Acharnon 17 Kifisia 14561 Greece; ^4^ IKERBASQUE Basque Foundation for Science Bilbao 48009 Spain

**Keywords:** adhesion, bioelectronics, DLP 3D printing, multi‐material printing, PEDOT:PSS

## Abstract

In this work, a new method of multi‐material printing in one‐go using a commercially available 3D printer is presented. The approach is simple and versatile, allowing the manufacturing of multi‐material layered or multi‐material printing in the same layer. To the best of the knowledge, it is the first time that 3D printed Poly(3,4‐ethylenedioxythiophene) polystyrene sulfonate (PEDOT:PSS) micro‐patterns combining different materials are reported, overcoming mechanical stability issues. Moreover, the conducting ink is engineered to obtain stable in‐time materials while retaining sub‐100 µm resolution. Micro‐structured bio‐shaped protuberances are designed and 3D printed as electrodes for electrophysiology. Moreover, these microstructures are combined with polymerizable deep eutectic solvents (polyDES) as functional additives, gaining adhesion and ionic conductivity. As a result of the novel electrodes, low skin impedance values showed suitable performance for electromyography recording on the forearm. Finally, this concluded that the use of polyDES conferred stability over time, allowing the usability of the electrode 90 days after fabrication without losing its performance. All in all, this demonstrated a very easy‐to‐make procedure that allows printing PEDOT:PSS on soft, hard, and/or flexible functional substrates, opening up a new paradigm in the manufacturing of conducting multi‐functional materials for the field of bioelectronics and wearables.

## Graphical abstract

This study introduces a new methodology for simultaneous multi‐material fabrication using a commercial 3D printer, enabling miniaturized bio‐shaped PEDOT:PSS structures on flexible substrates. Conducting bio‐shapes are combined with polymerizable deep eutectic solvents which enhance adhesion, ionic conductivity, and long‐term stability. The multi‐material electrode presents good skin adhesion, low skin impedance, and high signal‐to‐noise ratio, with excellent performance on forearm electromyography.

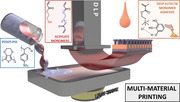

## 1 Introduction

Additive manufacturing (AM) has been announced as the 4th industrial revolution due to its customization degree and outstanding versatility.^[^ ^1^ ^]^ Metal printing,^[^ ^2^ ^]^ multi‐material printing, complex inks,^[^ ^3^ ^]^ or printing velocity are the remaining limitations that this technology still presents. Regarding multi‐material printing, AM should address the full control of the composition and geometry of each layer, resulting in a 3D‐printed structure with tuneable properties at the microscopic level. However, few examples in the literature address this challenge using a single‐step manufacturing approach (one‐go).^[^ ^4^ ^]^ One‐go multi‐material printing could be considered for printing methodologies in which the structure is not removed from the holder until all the materials are printed. As one example, J. A. Lewis and co‐workers^[^ ^5^ ^]^ pioneered the development of a new type of multi‐material 3D printing by an extrusion‐based method, which has been the gold standard processing technique up to now. This method is based on extrusion printing composed of a multi‐print head stage that is actuated by a bank of fast‐cycling pneumatic, enabling high‐frequency switching. Each syringe contains a different material, and the composition, function, and structure are tailored. This technique has been explored for manufacturing foldable origami patterns or soft robots that operate by air pressure, but also it has been used for adaptive 3D printing, where extrusion is performed onto arbitrary substrates.^[^ ^6^ ^]^ Lastly, in this method, the nozzle has been adapted for a rotational movement that enables programmable extrusion control over the local orientation of azimuthally heterogeneous features in multi‐material functional and structural filaments.^[^ ^7^ ^]^ Despite the versatility of extrusion‐based printing, the resolution is one of the biggest limitations compared with other techniques such as light‐based, laser, or ink‐jet printing. The main reasons are the minimum diameter of the nozzles available (100 µm) and the complexity of formulating inks with suitable shear‐thinning behavior that flows in such complex dynamics strains.^[^ ^8^ ^]^ Indeed, light‐based printing methods of 3D printing offer more precision and smaller feature sizes, but their implementation requires the assembly of complex setups, including fundamental parts of the printer such as motors, software, and controllers among others (see Table S1, Supporting Information). This degree of complexity includes tailored pathways to clean the ink after the sequential printing step or the activation of tailor‐made microfluidic holders.^[^ ^9^ ^]^ DeSimone et al.^[^ ^10^ ^]^ has developed a new technique based on continuous liquid interface production (CLIP) with feature resolution below 100 µm. CLIP uses an oxygen‐permeable window below the ultraviolet image projection plane, which creates a “dead zone” where photopolymerization is inhibited between the window and the polymerizing part, improving the working times of the printings from hours to minutes. As a step further, they introduced a continuous liquid through microfluidic channels on top of the dead zone. Through this mass transport control, injection continuous liquid interface technology (iCLIP) can readily pattern a single heterogeneous object with different resins in all cartesian coordinates.^[^ ^11^ ^]^ Chen Zen, Qi Ge, and co‐workers have reported the simplest multi‐material 3D printing approach that enables the fabrication of highly complex hybrid 3D structures. The 3D printing method consists of an iterative multi‐step process that comprises UV pattern printing followed by air‐jetting to remove the precursor solution residuals on the printed part. In contrast to extrusion‐based printing, their process possesses a maximum resolution of 100 µm and high fidelity. This approach has paved a new efficient way to fabricate soft devices and machines with greatly extended functionality.^[^ ^12^ ^]^ The same authors have modified the working principle from air‐jetting to radial centrifugation of the holder, cleaning the non‐polymerized acrylate residues.^[^ ^13^ ^]^ Using this methodology, the authors printed in a multi‐material approach using a material‐by‐material process an electroluminescent device composed of dielectric‐ZnO‐dielectric embedded in a hydrogel network.^[^ ^14^ ^]^ Lastly, Mayer et al.^[^ ^15^ ^]^ have presented a system based on a microfluidic chamber integrated into a state‐of‐the‐art laser lithography apparatus, scalable to seven different monomers and reaching pixel‐size structures. It is worth mentioning other less‐expanded light‐based multi‐material approaches with wavelength‐selective photocuring of interpenetrating polymer networks. The authors used green light selectively to initiate acrylate‐based radical polymerization, while shorter‐wavelength (blue) was used for the simultaneous formation of epoxy and acrylate networks. This strategy was presented as a promising and alternative orthogonal approach to conventional resins and photosensitizers.^[^ ^16^ ^]^

The second previously mentioned limitation in 3D printing is the lack of functional resins with tailored properties such as intrinsic conductivity,^[^ ^17^, ^18^ ^]^ hardness, elasticity, or bio‐resorbability.^[^ ^19^ ^]^ For instance, PEDOT:PSS, the most popular conducting polymer in bioelectronics, possesses flexibility, volumetric capacitance, and mixed ionic‐electronic conduction, rendering it ideal for bio‐recording. On the opposite to light‐based printing, extrusion‐based approaches of PEDOT:PSS have been engineered with co‐solvents such as dimethylsulfoside (DMSO) or polymers such as hydrophilic polyurethane, to obtain highly conducting inks^[^ ^20^ ^]^ or highly mechanically stable materials (over 400%, stretchability) and (over 3300 J m^−2^, fracture toughness).^[^ ^21^ ^]^ Other approaches based on polymer chemistry have tried to substitute the PSS polyelectrolyte to convert PEDOT into an intrinsically printable network. For example, 4‐styrene sulfonate (SS) and N‐(hydroxymethyl)acrylamide (NHMAA) were synthesized from block copolymers of PEDOT:P(SS‐co‐NHMAA) and extruded in multi‐material mode on polydimethylsiloxane (PDMS) substrates.^[^ ^22^ ^]^ In addition, PEDOT‐graft copolymers were synthesized with polyesters such as polylactic acid or polycaprolactone to design thermally‐driven printable conducting materials.^[^ ^20^, ^23^, ^24^ ^]^ However, 3D printing of PEDOT:PSS throughout light‐based approaches are still under development, due to limitations such as high absorption with reduced path length or the decreasing of the conductivity after its combination with other isolating polymers are the most remarkable issues.^[^ ^25^ ^]^ Up to date, only a few examples presented a PEDOT:PSS resin for use in digital light processing (DLP), bringing a formulation of this organic conductor with several acrylate monomers and polyethylene glycol acrylate (PEGDA) crosslinkers.^[^ ^26^, ^27^ ^]^

The last challenge to be addressed is the control of the geometry, for example throughout the formation of patterned topography, including probe diameter, height, tip shape, or bio‐shaped structures.^[^ ^28^ ^]^ The construction of this type of topography generally requires cleanroom facilities when feature sizes smaller than 100 µm are needed; however, the process could become tedious, slow, and costly. Indeed, the controllable fabrication of topographies with 3D printing could solve the tedious process. For example, the fabrication of bio‐mimicked gecko‐like protuberances, generally reported to gain dry adhesion, has only been done in cleanroom or lithographic pathways and has never been attempted through 3D printing, nor made of PEDOT conducting polymer.^[^ ^29^, ^30^ ^]^ One key example of doing a multi‐factorial combination of printing topographies with a conducting material is the demonstration of neural confinement and guidation of neurite outgrowth using a printed bio‐shaped micropillar.^[^ ^31^ ^]^ Apart from micropillars, other bio‐mimicked structures, such as mushroom‐like protuberances with the ability to switch wettability, reentrant, or generate self‐cleaned surfaces, could also be considered.^[^ ^32^, ^33^, ^34^, ^35^ ^]^ However, the main limitation with bio‐mimicking is a magnitude issue, where the target range is between 0.1 and 10 µm, while 3D printing approaches could only reach magnitudes of at least 10 times larger (≈50 µm). This mismatch of magnitudes makes the structure lose its inherent characteristic, that is, van der Walls forces. However, this limitation can be solved if the 3D printed structure is combined with other materials, using a polymer gel as an additive that confers the adhesion for example, overcoming the magnitude factor limitation. Indeed, mushroom‐like structures are particularly useful when combined with additives because their architecture is formed by a straight central pillar end‐capped with a rounded shape that becomes ideal for retaining a viscous gel within its structures.

In this paper, we addressed all the challenges described above using a commercial UV‐3D printer to create a complex device. First, we developed and characterized a water‐based conductive ink made of PEDOT:PSS with sub‐100 µm resolution capability and stability in its dry state. Then, we investigated multi‐material printed with mushroom‐like PEDOT:PSS protuberances on top of another flexible material, which confers flexibility to the whole device. The protuberances were combined with an acrylic quaternary ammonium monomer: a polymerizable pyrogallol‐based deep eutectic monomer (polyDES). The precursor deep eutectic monomer (DEM) is polymerizable throughout light and possesses high ionic conductivity due to its charged nature and adhesion properties thanks to its pyrogallol motif. Therefore, in our work, polyDES is deposited and photocured around the mushroom‐like structures, playing a dual role: adhesiveness and ionic conductor. The role of tridimensionality on the structure and the contribution of PEDOT:PSS mixed conduction was also addressed. The whole innovative concept was used to measure electromyography (EMG) in an adhesive self‐portable setup. Moreover, the material was printed and used 90 days later, evaluating stability in a dried state and demonstrating high performance. All in all, in this work, we present a change in the approach of 3D multi‐material printing, making it more accessible and versatile. This approach opens the technique to a multi‐factorial combination of polymers, designs, and additives to create unique complex architectures.

## 2 Experimental Section

### 2.1 Materials

All chemicals were used without any further purification. Poly(3,4‐ethylenedioxythiophene): poly(styrene sulfonate) (PEDOT:PSS) (PH1000, Heraeus Clevios), Poly(ethylene glycol) diacrylate (PEGDA, Sigma‐Aldrich, M_n_ = 700 Da), ethylene glycol (EG, Sigma‐Aldrich), [2‐(acryloyloxy) ethyl] trimethylammonium chloride solution (80 wt.% in water, Sigma‐Aldrich), 2,2‐Hydroxy‐4′‐(2‐hydroxyethoxy)−2‐methylpropiophenone (Irgacure‐2959, 97%, Sigma‐Aldrich), pyrogallol (PGA, Sigma‐Aldrich), 2‐hydroxyethyl acrylate (HEA, 97% Sigma‐Aldrich). The DLP commercially available inks, that is, GR 10 and *Formlabs Elastic FLELCL01* were purchased from and used without further modifications.

### 2.2 Methods

#### 2.2.1 PEDOT:PSS Ink

The photocurable conductive ink was prepared by mixing PEDOT:PSS aqueous solution (49 wt.%) with PEGDA (30 wt.%), HEA (20 wt.%), EG (1 wt.%), and 10 wt.% Irgacure 2959: 1 mg ml^−1^ riboflavin respect (1:1%v/v) to acrylate compounds. Finally, the mixture was ready to use after vortexing for 1 min.

#### 2.2.2 Preparation of Deep Eutectic Monomer (DEM)

The DEM was prepared following a previously reported protocol.^[^ ^36^ ^]^ The method consisted of mixing the ammonium salt monomer dissolved in water (80 wt.%) with PGA at 1:1 molar ratio under stirring at 70°C until obtaining a clear liquid.

#### 2.2.3 Synthesis of Polymeric Deep eutectic Solvent (polyDES)

The polyDES was obtained by photopolymerization of the DEM mixed with Irgacure 2959 (10 wt.%) as the photoinitiator. The DEM was poured into silicone molds or the mushroom‐shaped electrodes and UV‐irradiated for 5 min under UV light (385 nm, 30 mW cm^−2^). After a solid material was obtained, the sample was peeled off and stored at room temperature for posterior characterization.

#### 2.2.4 PEDOT:PSS Mushroom‐Shaped Electrodes

The mushroom‐shaped 3D‐printed electrodes were fabricated with an Asiga MAX X UV385. The procedure is detailed as follows: i) First, a rectangular flexible substrate of 32×11×0.5 mm was printed on the metallic holder using *Formlabs Elastic FLELCL01* resin. Parameter used provided by *Asiga* commanded by *Composer Inc*. software. ii) PEDOT:PSS/20% HEA‐co‐PEGDA was printed with three mushroom‐shaped shapes on top of the flexible substrate, covering the whole elastic material. For PEDOT:PSS/20% HEA‐co‐PEGDA, parameters were optimized as follows: 30 s were used as exposure time, 27 mW cm^−2^ as light intensity, 0 as X‐Y compensation, and 0.1 of Z‐compensation. The three mushroom‐shaped structures differed in diameter versus height of the protuberance, that is, planar, 1*vs*1, 1*vs*2, and 1*vs*3, maintaining the contact area constant. Afterward, the printed structure was washed gently with MiliQ water and unmounted from the holder using a metallic‐flexible spatula. Subsequently, the mushroom‐shaped electrode was dried with an air gun and placed inside a UV chamber for 5 min under UV light (385 nm, 30 mW cm^−2^). After that, the electrode was dried for 3 min at 50°C. Finally, the photocurable polyDES was drop cast on the electrode and homogenized along the whole pillars.

#### 2.2.5 Swelling

Squares of 1 mm^2^ were printed and dried at 25°C. Subsequently, the dry pieces were weighted (W_0_) and immersed in Milli‐Q water at 25°C. At established times, the samples were removed from Milli‐Q water, externally dried with filter paper to eliminate the excess water that could remain on the surface, and weighed (*W*_t_). The swelling percentage (*S*w) was calculated according to the following Equation (1):

(1) $$Sw=\frac{\left(W_{t}-W_{o}\right)}{W_{o}}\times 100$$

#### 2.2.6 Resistivity

The resistivity of the material was measured using a semiconductor device analyzer (Keysight B1500A) inside a Faraday cage under ambient conditions. Rectangular 10 × 5 mm lines were printed with 25, 50, 75, and 100 µm. Then, the resistivity was measured at the dried state and after swelling.

#### 2.2.7 Scanning Electron Microscopy

SEM images were taken on a ZEISS Gemini 300 VP scanning electron microscope using an acceleration voltage of 15 kV and an in‐lens secondary electron detector. SEM‐energy dispersive x‐ray spectroscopy (EDS) mapping was performed with an acceleration voltage of 15 kV at a working distance of ≈8 mm and 150x of magnification, using an Oxford Instruments EDS detector. The microwire was placed on top of carbon tape and analyzed in point‐by‐point scanning mode.

#### 2.2.8 Lap‐shear Test

The adhesion properties of the mushroom‐shaped/polyDES electrodes were evaluated at room temperature using a Tinius Olsen 1ST instrument and applying a perpendicular force to the adhesive bond with a displacement rate of 100 mm min^−1^. The adhesion was measured against a Kapton substrate. Three different mushroom‐shaped height structures were compared with the planar electrode. The adhesive contact area was maintained constant for all the specimens (297 mm^2^). The tests were performed on at least three samples for each electrode height to determine the average lap‐shear strength, and the error was calculated as the standard deviation of the measurements.

#### 2.2.9 Shape Fidelity

The fidelity of the printed structures was measured under the optical microscope (Olympus IX 71 microscope) where *R* is the experimental size of features measured and *R*o is the real size of the same features as designed in Autodesk Inventor. The microscope was calibrated before measurements.

#### 2.2.10 EMG Recording

All the EMG recording experiments were performed after approval of the Ethics Committee of the Department of Engineering at the University of Cambridge (6/9/2018, IONBIKE) and after obtaining informed consent from participants. The measurements were recorded using an RHS stimulation and recording system (Intan Technologies) at a 30 kHz sampling rate. Testing areas were gently wiped with a tissue wet in ethanol for skin preparation. Two different recording locations were selected for this work, that is, the inner forearm and hand thumb region. A commercial Ag/AgCl electrode (MLA 1010B, ADInstruments) was placed on the elbow for voltage reference. Recorded EMG signals were filtered using a 50 Hz band‐stop filter and a band‐pass filter with 10 and 400 Hz cut‐off frequencies. The signal‐to‐noise (SNR) ratio was calculated as the root mean squared (RMS) ratio of the measured voltage during muscular activation normalized by the number of samples, divided by the RMS of the signal measured, and normalized by the number of samples. The envelopes for each EMG were generated by means of a moving average filter with a window length of 50 000 samples. Envelope variability was calculated from the histogram of magnitude values during muscle contraction across the shown trial for each material. The histogram fitting represents a Gaussian distribution.

#### 2.2.11 Fourier Transform Infrared Spectroscopy (FTIR)

FTIR was employed to characterize the structure of the dried composites, the DEM, and the PEDOT:PSS. A Nicolet Magna 6700 spectrometer was used to acquire the spectra in the range of 400–4000 cm^−1^ with a 4 cm^−1^ resolution and averaged over 24 scans.

#### 2.2.12 Photopolymerization Reaction Kinetics

The photopolymerization reaction conditions were studied by FTIR and photorheology. FTIR spectra were recorded in the attenuated total reflectance (ATR) mode in a Thermo scientific model Nicolet 6700 FTIR spectrometer, with a resolution of 4 cm^−1^, mirror speed of 0.3165, and 5 scans. Samples were placed in a zinc selenide glass, and ATR‐FTIR spectra were recorded at room temperature every 6.48 s by exposing the photocurable inks to UV light (wavelength = 390 nm, power = 30 mW cm^−2^) for 5 min. The conversion was calculated with Equation (2) by measuring the area of the peaks located at 1417 cm^−1^, which correspond to the C = C stretching vibrations:

(2) $$C=C\;\mathrm{conversion}\;\left(\%\right)=\left(1-\frac{A_{t}}{A_{0}}\right)\times 100$$

where *A*t is the area of the band at a time *t*, and *A*_0_ is the area of the band at zero time.

#### 2.2.13 Photorheological Measurements

Photorheological measurements were carried out at room temperature in an AR‐G2 rheometer (TA Instruments) using a UV‐light lamp (wavelength = 365 nm, power = 30 mW cm^−2^), oscillation stress of 100 Pa, and 2 Hz frequency. The gel point was determined by placing the samples on a glass parallel plate of 20 mm diameter, letting them stabilize for 60 s to be subsequently irradiated for 30 s, and continuing to register the elastic modulus (G′) and loss modulus (G′′) until a plateau was reached.

#### 2.2.14 Electronic Conductivity

The electronic conductivity of the dried samples was measured in four‐point probe mode using a Keysight B1500A apparatus. Three measurements were obtained, and the average value was reported, with its corresponding deviation standard error.

#### 2.2.15 Ionic Conductivity

The ionic conductivity was measured by electrochemical impedance spectroscopy (EIS), employing an Autolab 302N potentiostat‐galvanostat coupled with a Microcell HC station, which provided temperature control. The polyDES was measured by being deposited inside a silicon mould of 8 mm intern diameter and ≈1.245 mm thickness. EIS measurements were carried out from 85 to 25°C, with a step of 10°C each, holding the temperature for 10 and 20 min before each temperature change to allow temperature equilibrium in the whole sample. The frequency range was varied from 105 to 1 Hz, with 10 mV amplitude.

## 3 Results and Discussion

### 3.1 PEDOT:PSS Ink for Multi‐Material and High‐Resolution Stereolithographic 3D Printing

**Figure 1:**
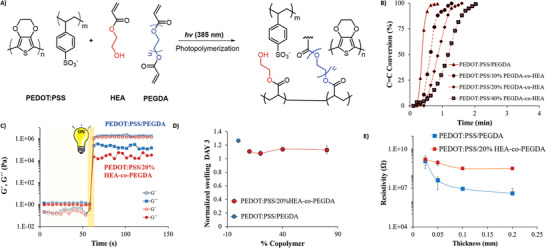
A) Photopolymerization scheme of PEDOT:PSS ink that occurs within the DLP printer when irradiated. PEGDA and HEA react throughout a radical polymerization initiated by the photoinitiators, creating an interpenetrated network with PEDOT:PSS, B) Evolution of the C═C conversion versus time during the photopolymerization depending on HEA and PEGDA composition, C) Rheological measurements of the elastic modulus (G') and loss modulus (G") to determine the gel point when the inks are irradiated for 5 s (in the interval from 60 to 65 s), D) Normalized swelling after 3 days uptaking water of PEDOT:PSS/PEGDA and PEDOT:PSS/20% HEA‐co‐PEGDA and E) Resistivity of PEDOT:PSS/PEGDA and PEDOT:PSS/20% HEA‐co‐PEGDA as a function of the thickness of the structures.

In order to develop a conductive ink for high‐resolution structures, PEDOT:PSS was formulated within a photocurable matrix composed of polyethylene glycol diacrylate (PEGDA) as a crosslinker and a mixture of riboflavin/Irgacure‐2959 as the photoinitiators (see **Figure** **1**A). Irgacure‐2959 major drawback is its limited molar absorptivity at wavelength 365 nm; therefore, we combined it with riboflavin as a sensitizer, increasing the absorption wavelength and molar coefficient absorption, thus improving the formation of radicals under light exposure.^[^ ^9^, ^37^ ^]^ The photopolymerization kinetics were studied by ATR‐FTIR. The peak located at 1417 cm^−1^, which corresponds to the ‐C═C‐ out‐of‐plane bending vibrations, disappears as the photopolymerization reaction proceeds (Figure S1, Supporting Information). The use of riboflavin has been previously described for fast visible‐light‐induced photopolymerization of acrylic monomers in the presence of *π*‐conjugated systems in water media.^[^ ^38^ ^]^ Recently, Gallastegi and co‐workers have proven the role of PEDOT:PSS as an accelerator of visible‐light photopolymerization reaction. The reaction used a type‐II precursor composed of triethanolamine and riboflavin, resulting in conductive hydrogels.^[^ ^27^ ^]^ In this regard, PEDOT:PSS/PEGDA reaches 92% of conversion after 30 s of irradiation when a mixture of sensitizer and initiator is employed. The conversion reaches the highest value when 10 wt.% Irgacure‐2959:riboflavin (1:1%w/w) is used and decays dramatically to 30% for 5 wt.%. The absence of polymerization is observed when Irgacure 2959 is not added to the ink. PEDOT:PSS is a highly absorptive dispersion with a reduced path‐length transmission in the UV–vis‐NIR spectra.^[^ ^39^ ^]^ Hence, higher concentrations of PEDOT:PSS reduce the polymerization rates, hindering the formation of self‐supported tridimensional structures. The optimal ratio between PEDOT:PSS and the acrylate monomers is fixed at 50/50%w/w for the formulation of the inks. This data is in accordance with other reported two‐photon polymerization (2PP) or DLP approaches of PEDOT:PSS.^[^ ^40^, ^41^ ^]^ Photo‐rheology was used to track the viscoelastic behavior of the hydrogel network after applying UV light irradiation. After UV exposition for 5 s (Figure 1C), the gel reaches a plateau with storage and loss moduli of 1 × 10^6^ Pa, demonstrating the capability of the inks to achieve the gel point before getting a complete conversion. This photopolymerization allows us to obtain 3D architectures very fast and is optimum for 3D printing.

Different comonomers were formulated with PEGDA to reduce the crosslinking degree and improve the mechanical properties, for example, ethylene glycol phenyl ether methacrylate, ethylene glycol methyl ether methacrylate, and 2‐hydroxyethyl acrylate (HEA). The most hydrophobic monomers bearing phenyl or methyl pendant groups presented phase separation when polymerized in the presence of PEDOT:PSS. We hypothesized that this process occurs due to the insolubility of the polymers formed in water media. In contrast, the hydroxyl‐terminated monomer, HEA produced an interconnected network in different percentages without phase separation. However, including HEA monomer in the reaction decreases the polymerization rates. Figure 1B shows the impact of HEA in the kinetics of the photoreactions, 10, 20, and 40% of HEA increased to 1, 1.5, and 2 min, respectively, the time to reach 90% of monomer conversion. In addition, the photo‐rheology indicates that HEA units did not significantly affect the elastic modulus (G') of the hydrogel network, which remained at about 1 × 10^6^ Pa. (see Figure 1C). However, PEDOT:PSS/20%HEA‐co‐PEGDA hydrogels reduced its swelling to 20% compared with the homopolymer of PEDOT:PSS/PEGDA (Figure 1D). This effect could be explained by the decrease in the hydrophilicity of the network, driven by the inclusion of short hydroxyethyl pendant groups (HEA) instead of long polyethylene glycol chains (PEG700).^[^ ^42^ ^]^ Figure S2 (Supporting Information) shows the compressive stress‐strain curves of PEDOT:PSS/20% HEA‐co‐PEGDA. The Young's modulus of the copolymer is 13.4 ± 0.2 MPa which is lower than the homopolymer PEDOT:PSS/PEGDA (35.4 ± 1.4 MPa) elsewhere reported.^[^ ^40^ ^]^ For both materials, the resistivity measured by a four‐point probe (4PP) is in the same range depending on the thickness, that is, 3.91 MΩ for PEDOT:PSS/PEGDA or 3.25 MΩ PEDOT:PSS/20% HEA‐co‐PEGDA, respectively (Figure 1E). It is well known that the dilution of PEDOT:PSS in a polymer matrix without additives decreases the conductivity, as reported in similar (meth)acrylate‐based systems.^[^ ^40^, ^43^ ^]^ UV–vis spectroscopy was performed to compare the PEDOT:PSS oxidation state obtained within a complex matrix composed of insulating polymers, PEGDA, and PEGDA‐co‐HEA (Figure S3, Supporting Information). Hence, PEDOT:PSS dispersion was compared with photo‐polymerized PEDOT:PSS/20%HEA‐co‐PEGDA, demonstrating the typical *π*–*π* ^*^ PEDOT absorption bands at ≈600–800 nm.^[^ ^44^ ^]^ This fact confirms that despite the PEDOT:PSS being surrounded by a non‐conductive polymer matrix, the electroactivity of this organic conductor and its bipolaron state is still present.

### 3.2 3D Multi‐Material Printing of PEDOT:PSS in One‐Go

**Figure 2:**
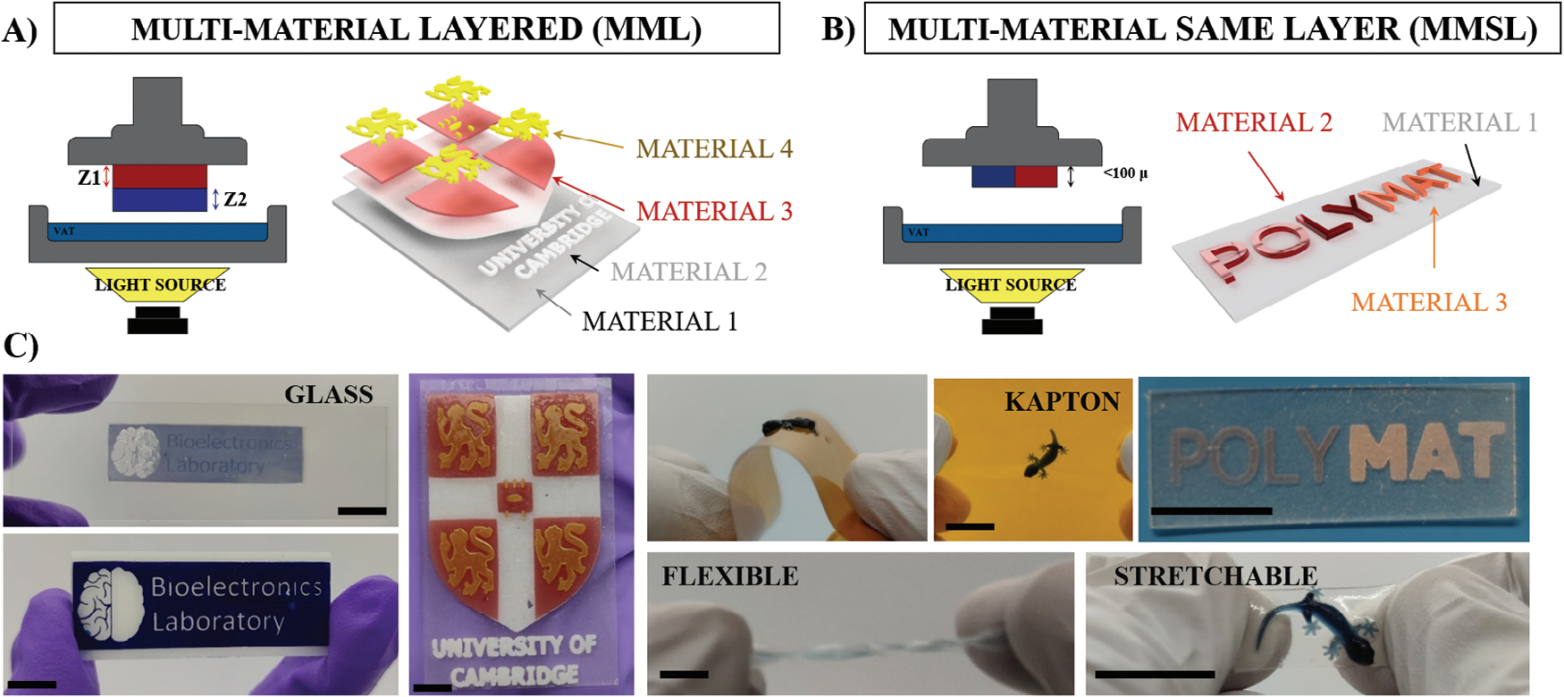
Illustration of the DLP‐based multi‐material 3D printing in different layers MML A), same layer MMSL B,C) PEDOT:PSS/PEGDA ink is printed on top of glass coverslip, on top of kapton film, and multi‐layered on top of a flexible or stretchable material, demonstrating the possibility to create highly stretchable and flexible complex multi‐materials (scale bar: 1 cm).

**Figure** **2** describes the overall concept of multi‐material printing here presented. The method is straightforward and performed on a commercially available 3D printer, where the printing mechanism relies on the conventional “bottom‐up” projection. The digitalized UV light is irradiated from the UV projector, placed at the bottom of the printing platform, and the holder moves vertically to control the thickness of each slice. Compared to the other multi‐material printing approaches reported in the literature, this method is the only one where the holder is removed, cleaned of non‐react residues, and placed again to perform the printing. In previous works, researchers have previously reported complex adaptations of the 3D printers with air or radial movements.^[^ ^7^, ^12^, ^13^ ^]^ Moreover, this methodology allows a more controllable Z‐position in each printing step. In our multi‐material approach, two types of printings could be performed: Multi‐materials in different layers (**MML**) or multi‐materials in the same layer (**MMSL**).

In order to describe the mechanism, *Pro3Dure GR10* resin was colored and used for the illustration of the multi‐material fabrication. The two printing mechanisms could be described in the following steps (see Figure 2A): (i) 3D printing of the red material with a specific shape and height (Z1), the printing is performed on top of the 3D printer holder, (ii) manual exchange of the polymeric VAT with a second material, (iii) adjust the new height within the software to the previous height (Z1), (iv) 3D printing of the blue material, on top of the previous (red) material (**MML**). If the previous printing is smaller than 100 µm in height, printing is also feasible in the same layer (**MMSL**) (Figure 2B).

Thanks to this technique, apart from the colorful complex **MML** or **MMSL** structures, as illustrated in Figure 2A‐B, it is also possible to 3D print PEDOT:PSS structures on top of stretchable or flexible substrates or specific substrates such as glass, Kapton, or Parylene‐C^[^ ^45^ ^]^ (Figure 2C). This easy‐to‐make procedure allows printing PEDOT:PSS on functional substrates, opening a new approach in manufacturing conductive multi‐functional materials. For example, PEDOT:PSS miniature topographies were DLP printed on a commercial 3D elastic resin (*Formlab FLECLC1*), making a flexible material. Then, the substrate material was cleaned mechanically, and the VAT was changed to the PEDOT:PSS‐ink. Finally, the PEDOT electroactive ink was printed on top of the base material, demonstrating satisfactory small feature size and mechanical stability. It is worth mentioning that PEDOT:PSS/PEGDA structures smaller than 200 µm cannot be printed and removed from the holder if printed without a base material, because of the poorer mechanical properties of the acrylate structure when miniaturized (Figure S4, Supporting Information).

### 3.3 Bio‐Shaped Electrodes

The optimal printing conditions were investigated by DLP of 3D patterns with reliefs and holes at different irradiation times (5, 10, 20, and 30 s) and layer heights (0.025, 0.05, 0.1, and 0.2 mm) using the photopolymerizable ink PEDOT:PSS/PEGDA (Figure S5A–D). When low irradiation times were employed (<10 s), the printing patterns did not show the designed structure because the ink could not be successfully photocured, as proven by the low monomer conversion (<30%). Increasing the exposure time to UV light by up to 30 s gave rise to patterns with better printing resolution. It was observed that lower layer thickness (0.025 and 0.050 mm) did not result in significant changes, while 0.050 and 0.100 mm introduced more variability in the printings. Therefore, 30 s of exposure time and 0.025 mm of layer height were chosen as the optimal parameters that afford accurate photopolymerization rates.

**Figure 3:**
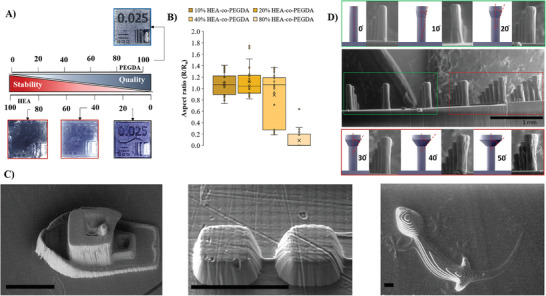
A) Scheme and B) Aspect ratio (R/Ro) quantification of the PEDOT:PSS/20% HEA‐co‐PEGDA formulation. The way that HEA is included in the copolymer on the quality versus mechanical stability can be observed, C) SEM images (from left to right: boat, squares, and alligator) of different sub‐100 µm examples for the PEDOT:PSS/20% HEA‐co‐PEGDA printing (scale bar: 100 µm) and D) SEM of PEDOT:PSS/20% HEA‐co‐PEGDA with different angle‐test topographies. The image shows the 3D printed structures with different angles of the Mushroom's protuberances versus those designed by Autodesk.

However, when structures made of PEDOT:PSS/PEGDA dry out, they collapse and lose their shape due to a combination of the forces produced on the base material and the aqueous nature of PEDOT:PSS dispersions. This effect occurs due to the high degree of crosslinking, as only PEGDA is used as the acrylate monomer. In this line, the copolymer PEDOT:PSS/HEA‐co‐PEGDA presented higher time stability than the PEDOT:PSS/PEGDA homopolymer, as incorporating hydroxyethyl pendant groups increases the network's flexibility, reducing internal tensions during drying and preserving the structures' integrity. Nevertheless, **Figure** **3**A,B demonstrates the impact on printing resolution when adding HEA to the ink. In accordance, the optimal formulation that balances printability versus high resolution is PEDOT:PSS/20%HEA‐co‐PEGDA. As can be observed in Figure 3C, this optimized formulation and printing conditions allowed for sub‐100 µm resolution, resulting in outstanding feature sizes, such as a cube smaller than 70 µm or the chimney of the boat. We used this capability to build an electrode bio‐shaped by a mushroom's protuberances system, which consists of a monolithically shaped column with a mushroom‐shaped termination. The design of the structure, allows it to retain the gel. Previously, this type of mushroom‐like termination has been demonstrated to improve dry adhesion, friction contribution, and suction effect and enhance the retention of a viscous liquid.^[^ ^32^ ^]^ Some of the shaped‐design reported possessed a 90° angular shape end‐capped, improving the aforementioned retention effect. However, DLP printing is a bottom‐up approach with digital projections of each layer, so it cannot build structures with more than 50° in respect to the Z‐axis. Figure S6 (Supporting Information) and Figure 3D show an angle test used to compare the real versus the designed appearances of the protuberances studied by SEM. Protuberances manufactured with angles of 50° result in mushroom‐like appearances, that are the closest match to the theoretical design. Like the angle test, the density test was performed to optimize the maximum number of protuberances per area, improving the mechanical stability of the electrode.

**Figure 4:**
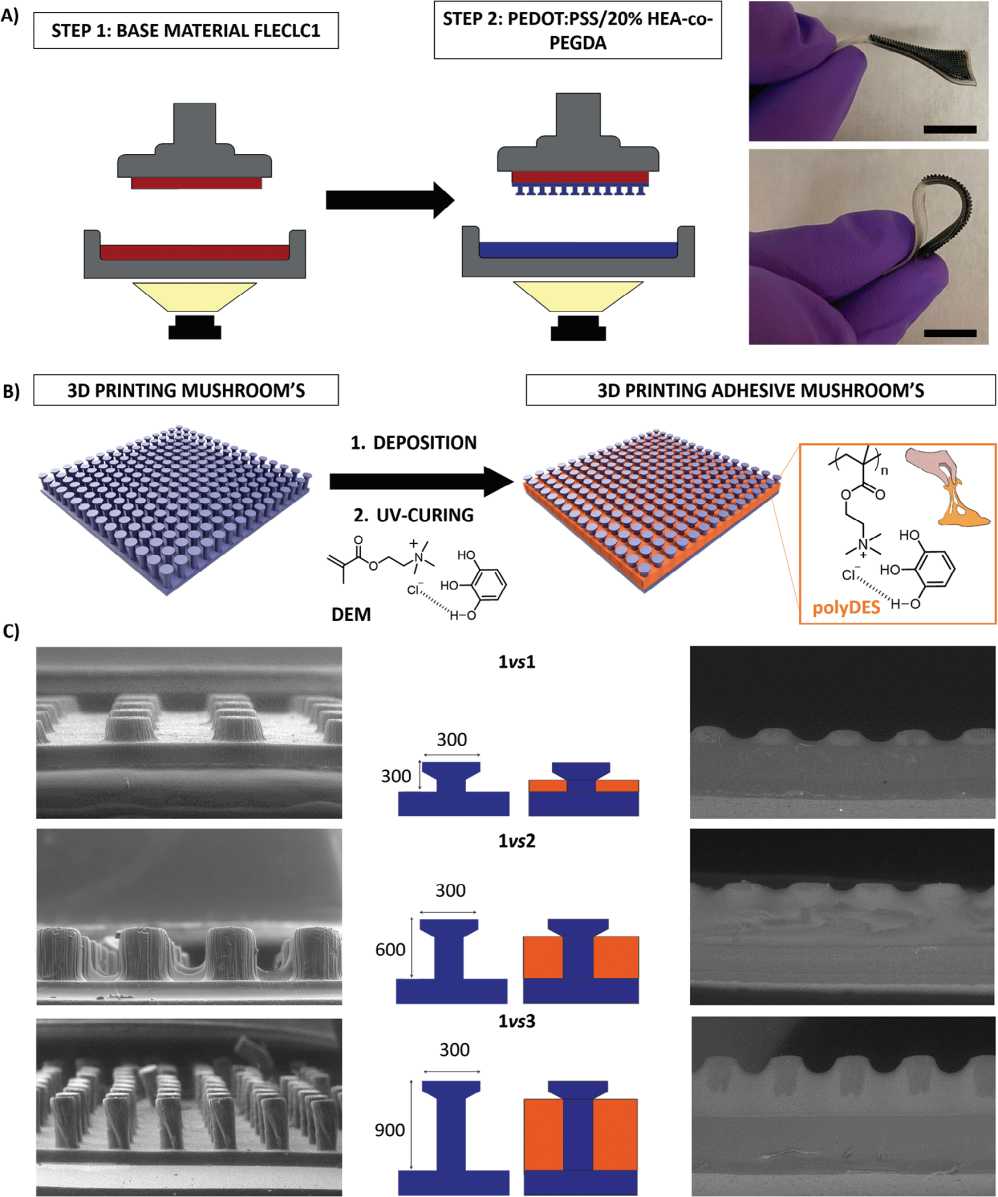
A) Left: Scheme of the printing process of a PEDOT:PSS bio‐shaped electrode. First, a flexible resin (FLECLC1) is printed, and then, PEDOT:PSS/20% HEA‐co‐PEGDA resin is printed forming mushroom bio‐shapes. Right: An example of FLELCL01/PEDOT:PSS/20% HEA‐co‐PEGDA appearance and flexibility is shown in the photo (scale bar: 1 cm), B) Schematic representation of the formation of PEDOT:PSS/20% HEA‐co‐PEGDA Mushroom's shaped materials. The DEM is deposited by drop cast on the printed topographies and then cured for 5 min under UV light (365 nm) and C) SEM images of the conducting mushroom without (left) and with (right) the DEM.

The application of our multi‐material approach to the bioelectronics field consisted of the fabrication of conducting mushroom‐shaped electrodes. For such purpose and considering the mentioned limitations, PEDOT:PSS/20%HEA‐co‐PEGDA was printed on a flexible material (Formlab FLECLC1), improving the stability, interfacial contact, and interfacial stress fracture. Figure 4A shows the printing steps: 1) FLECLC1 was printed on the printer holder and a new Z‐limit with the height of the printing was set and 2) PEDOT:PSS/20% HEA‐co‐PEGDA was printed on top of FLECLC1 with a mushroom bio‐shape. The final device is highly flexible and conformable on the skin, bending on itself without delamination or breaks. Moreover, the mushroom bio‐shaped devices were combined with functional additives that could offer more complexity to the architecture, bringing multiplexing properties. In the selection of functional additives, deep eutectic solvents (DES) appear as a new class of ionically conductive materials that improve the properties of mixed conduction of PEDOT:PSS.^[^ ^46^ ^]^ Particularly, deep eutectic monomers (DEM) and their polymers (polyDES) made of bio‐based polyphenols, form strong hydrogen bonds, creating tough adhesives while the polymer is composed of charged species, rendering it ionically conductive.^[^ ^36^ ^]^ Figure 4B shows the structure of the pyrogallol‐based DEM balanced with trimethyl ammonium chloride methacrylate monomer. Pyrogallol is a natural polyphenol found in many plants and has been broadly used in biomaterials for tissue adhesives.^[^ ^47^, ^48^ ^]^ In particular, a recent study demonstrated that pyrogallol is compatible with the human cell line HEK293 (human embryonic kidney), which makes ideal for ski‐contact applications.^[^ ^49^ ^]^ The PEDOT:PSS/20% HEA‐co‐PEGDA mushroom‐shaped electrodes consisted of 266 protuberances with exact topography where the DEM was drop‐casted and then photocured around the protuberances, conferring a hybrid device with adhesion and mixed ionic‐electronic conductivity (see Figure 4B).

The appearance of a real device before and after the drop‐casting of polyDES is observed in Figure S8A (Supporting Information). Hence, three electrodes with different aspect ratios (i.e., the diameter of the protuberance vs height) were printed and compared. Accordingly, the mushroom protuberances are 1*vs*1 (300 vs 300 µm), 1*vs*2 (300 vs 600 µm), and 1*vs*3 (300 vs 900 µm). The amount of DEM deposited around the protuberances was calculated using the Autodesk design, aiming to fill the volume of the empty electrodes, that is, 18, 50, and 81 µl cm^−2^ for 1*vs*1, 2, and 3, respectively. Figures 4B and S7 (Supporting Information) shows the electrode appearance pre‐ and post‐covered with the DEM. Moreover, PEDOT:PSS/20% HEA‐co‐PEGDA/polyDES 3D printed protuberances are consistent with the design structure, and SEM images of the PEDOT:PSS/20% HEA‐co‐PEGDA confirm the volume used. SEM images also show that the combinational design of protuberances and the mushroom terminations are optimum to retain the DEM first and the polyDES after curing, avoiding the complete covering of the structure and interfering with the geometry of the electrode (see Figure S8A, Supporting Information). SEM‐EDS also confirmed the presence of only residual chlorine elements on the top of the mushroom pillars, indicating that the deposition did not cover the mushroom and maintained the scope of conferring tridimensionality to the polyDES deposition. (see Figure S8B, Supporting Information).

**Figure 5:**
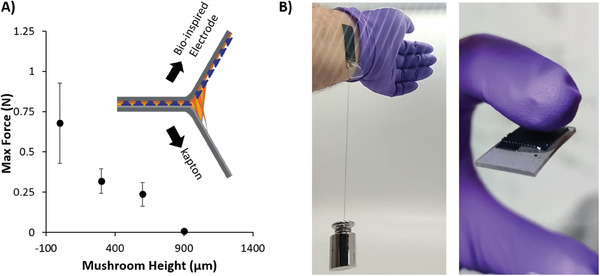
A) Evolution of the lap‐shear strength as a function of the different protuberance heights of the mushroom‐shaped electrode and B) Photos of 1*vs*2 PEDOT:PSS/20%HEA‐co‐PEGDA/polyDES mushroom‐shaped electrodes holding a 0.5 kg weight and showing stickiness to a polymeric material.

This polyDES is based on the combination of a natural polyphenol and an acrylic quaternary ammonium monomer, producing a viscous liquid‐like ionic polymer that is extremely sticky with an adhesive stress of around 1.25 MPa.^[^ ^36^ ^]^ Gallol adhesion is attributed to chemical interactions between phenolic groups and the neighboring substrate, including hydrogen bond and *π*–*π* interactions.^[^ ^50^ ^]^ The mechanism of adhesion between a gallon moiety and the skin has been suggested as a partial deprotonation of gallon functional groups in physiological conditions that convert to reactive quinone groups, which further react with thiol and amine terminals of biomacromolecules present in the tissue.^[^ ^51^, ^52^ ^]^ The adhesive properties of all mushroom electrodes were evaluated through the lap‐shear test using the mushroom‐shaped electrode coated with the polyDES on kapton foil, with both faces remaining parallel at the beginning of the measurement. Then, by the action of a perpendicular force, both faces started to peel‐off, while the force and the displacement were being monitored. (see **Figure** **5**A, inset)

We observed an adhesiveness factor influenced by the height of the protuberances and, therefore, the amount of polyDES. Figure 5A shows the dependence of the maximum adhesion force if compared with the height, being 0.67 ± 0.25 N for the planar substrate and 0.312 ± 0.1, 0.24 ± 0.1, and 0.01 ± 0.001 N for 300, 600, and 900 µm, respectively. It has been suggested in the literature that this phenomenon occurs because thicker thickness can lead to a decreased ability of the adhesive to effectively transfer stresses between the polyDES and the kapton, potentially resulting in premature joint failure.^[^ ^53^ ^]^ Figure S9A (Supporting Information) shows the shear‐lap test; the planar substrate presents a Gaussian‐type adhesion curve, indicating higher shear strength but very low deformability. However, 1*vs*1 and 1*vs*2 PEDOT:PSS/20% HEA‐co‐PEGDA present more elongated curves, with non‐linear behaviors producing large inelastic deformations with lower shear strengths. The process of peeling off occurs more irregularly when patterning the adhesion area, as well as the thickness of the adhesive material.^[^ ^54^, ^55^ ^]^ Adhesion forces of the 1*vs*2 PEDOT:PSS/20%HEA‐co‐PEDOT were enough to hold and lift a weight of ≈0.5 kg (Figure 5B). Moreover, the surface morphology of the used mushroom electrode was observed under scanning electron microscopy (SEM) before and after being in contact with the skin (Figure S9B, Supporting Information). After skin contact, the polyDES remains in the electrode protuberances, indicating that the adhesive mechanism does not leave residues that could potentially produce skin irritation.

### 3.4 Electromyography (EMG) Measurements

**Figure 6:**
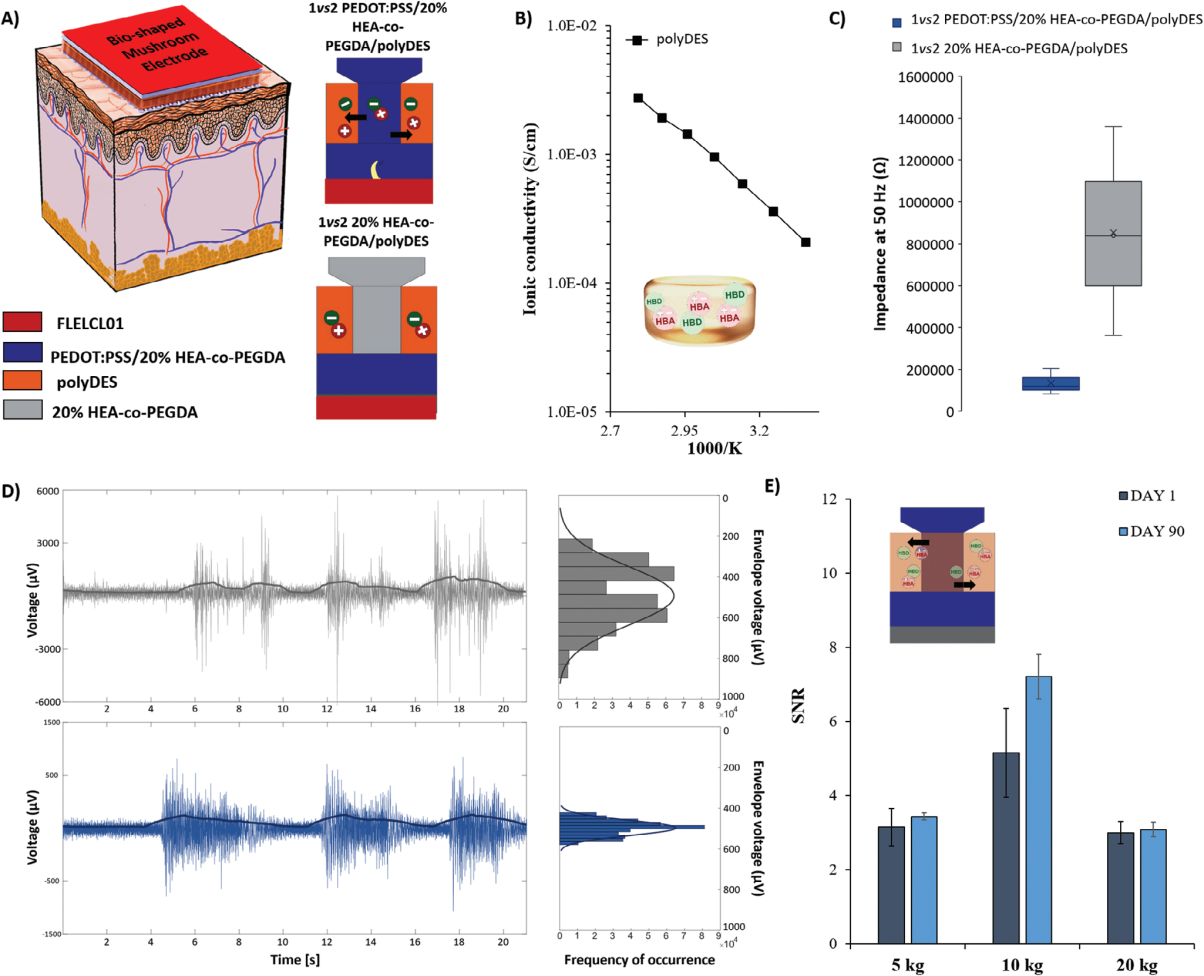
A) Left: Scheme of the mushroom‐shaped electrode adhered on the skin for epidermal bio potential detections of electromyography (EMG), Right: Schematic representation of the different multi‐material printed electrodes, B) Ionic conductivity of polyDES conducted in a sandwich‐like cell by EIS at different temperatures, C) Comparative impedance values of 1vs2 mushroom's electrodes on the skin at 50 Hz with and without PEDOT:PSS, D) Left: 22 s of EMG recordings on the forearm of 1*vs*2 PEDOT:PSS/20%HEA‐co‐PEGDA/polyDES and 1*vs*2 20%HEA‐co‐PEGDA/polyDES electrodes when 5 kg of grip were applied. The power spectrum is plotted in continuous blue or gray lines on the EMG data. RIGHT: Histogram and fitted signal with a Gaussian distribution of the amplitude signal when the muscle is activated and E) Signal‐to‐noise ratio (SNR) of 1vs2 PEDOT:PSS/20%HEA‐co‐PEGDA/polyDES at day 1 and after 90 days, demonstrating the high stability of the electrodes.

**Figure** **6**A shows the final geometry scheme adhered on the skin for EMG recordings. The stickiness of the material enhances the bio‐potential signal recording of muscle groups for electromyography (EMG), as demonstrated previously.^[^ ^56^ ^]^ EMG is a technique that records muscle activation and relaxation versus signal amplitude depending on motion. The most useful and important EMG frequencies range from 10 to 400 Hz, and generally, 50 Hz is used to compare the performance of electrodes. EIS was also investigated on the skin using a two‐electrode configuration. All the samples displayed a frequency‐dependent response, showing high impedance values at lower frequencies. (see Figure S10A, Supporting Information). In addition, the range of impedance values was in the same order of magnitude as a commercial Ag AgCl^−1^ electrode. Apart from the adhesiveness, the mushroom‐shaped structure combined with the polyDES confers an ionic conductivity‐dependant on the mentioned tridimensionality. The impedance value was correlated to the height of the device, as higher protuberances are expected to accumulate more polyDES, gaining ionic conductivity and reducing the impedance of the skin interface. Indeed, the mushroom‐shaped 1*vs*3 PEDOT:PSS/20%HEA‐co‐PEGDA/polyDES electrodes showed the lowest impedance values (113 ± 55 kΩ), followed by 1*vs*2 (125 ± 62 kΩ) and 1*vs*1 (580 ± 114 kΩ) configurations. In comparison, wet Ag AgCl^−1^ electrodes showed impedance values of 80 ± 32 kΩ (see Figure S10B, Supporting Information).^[^ ^57^ ^]^ The impedance value can also be influenced by the adhesion quality, the electrode's chemical composition, and the subject's skin. Moreover, a key aspect of minimizing impedance is to reduce the disparity between bio‐conduction using ions and conduction using electrons. To address the performance and role of each part of the device, that is, the role of polyDES and PEDOT:PSS mushroom‐shaped pillars, we selected a trade‐off configuration that possesses good adhesiveness and reduces the skin‐impedance interface. This electrode configuration was: 1*vs*2 PEDOT:PSS/20% HEA‐co‐PEGDA/polyDES.

First, we decipher the effect of the polyDES in the EIS on the skin. For instance, 1*vs*2 PEDOT:PSS/20% HEA‐co‐PEGDA was compared against 1*vs*2 PEDOT:PSS/20% HEA‐co‐PEGDA/polyDES. Figure S11A (Supporting Information) shows that the uncoated 1*vs*2 PEDOT:PSS/20% HEA‐co‐PEGDA electrode has an impedance value of 125 ± 62 kΩ while the polyDES‐coated electrode possesses 179 ± 28 kΩ, pointing out that the value of impedance does not present a significant impact at lower frequencies for the same configuration. However, the inclusion of the polyDES in the electrode amplified the capacitive effect at low frequencies, increasing the phase angle from *φ* ≈24 ± 17° to 56 ± 4° (Figure S11B, Supporting Information). The ammonium charges of the acrylate moiety counterbalanced with chlorine anions and its hydrogen bond interaction with the phenolic groups of the pyrogallol can store charges. This storage ability enhances the propagation of EMG bioelectronic signals from the muscle to the skin‐electrode interface, where they are then sensed as motion of charges. Indeed, the use of ion‐gels composed of cholinium cation with choline‐based salts as ionic liquids in combination with polyphenols as tannic acid and polyvinyl alcohol (PVA)^[^ ^58^ ^]^ or gelatin,^[^ ^59^ ^]^ has been successfully demonstrated for electrophysiological measurements of EMG recordings. The materials demonstrated excellent biocompatibility, decent biopotential recording, and improved long‐term stability despite the absence of mixed ionic‐electronic conduction.^[^ ^60^ ^]^ In order to calculate the ionic conductivity, the EIS of the polyDES was measured between two metal electrodes. The ionic conductivity of the polyDES goes from 2.09 to 10^−4^ at room temperature to 2.78 · 10^−3^ S cm^−1^ at 85°C (see Figure S12A, Supporting Information). This ionic conductivity (σ_ionic_≈10^−4^ S cm^−1^) value is comparable to homologous iongels with lower biocompatibility due to the presence of bis(trifluoromethanesulfonyl)imide salts.^[^ ^61^ ^]^ Moreover, the capacitive behavior of the material can be observed by the sloped line of the impedance and the high phase value (*φ* ≈ −70°) (see Figure S12B, Supporting Information).

Once we determined the role of the polyDES, we wanted to decipher the contribution of the PEDOT:PSS in the mushroom's protuberances. For such purpose, we used our approach of multi‐material printing and manufactured two different types of electrodes: one with PEDOT:PSS as the mixed ionic‐electronic conductor at the interface with the skin and another one without the mixed conductor and 20%PEGDA‐co‐HEA polymer instead (**Figure** **6**A). Electronic and ionic conductivities of dry PEGDA‐composed materials are very low (≈1.10^−9^ S cm^−1^); therefore they can be considered non‐contact dry electrodes for EMG recording. This electroes types produces high levels of sensitivity to motion artifacts and time‐dependent stray charges.^[^ ^62^ ^]^ On the opposite, as Robert Howe pointed out, the presence of a mixed ionic‐electronic conductor as PEDOT:PSS at the interface with the skin minimizes the skin‐electrode impedance and increases the signal‐to‐noise ratio.^[^ ^60^ ^]^

The tendency of the skin impedance for the same electrode setup, 1*vs*2, with and without PEDOT:PSS was first evaluated. Figure 6C shows a decrease in the skin impedance value almost five times from 620 ± 499 to 126 ± 74 kΩ when PEDOT:PSS is used as a mixed ionic‐electronic conductor at the interface (Figure 6C). Then, the performance of the electrodes for EMG recordings at three different grip forces, that is, 5, 10, and 20 kg were compared (see Figures S13,S14, Supporting Information). Figure S15 (Supporting Information) shows the SNR of 1*vs*2 mushroom electrodes with and without PEDOT:PSS for 5, 10, and 20 kg. At first galance, PEDOT:PSS increase the SNR from 9.10 ± 0.28 to 10.40 ± 1.45 for 5 and 20 kg, respectively. Meanwhile, the 1*vs*2 mushroom electrodes without PEDOT:PSS decrease from 7.54 ± 1.70 to 0.98 ± 0.97 for the same comparison of weights. Moreover, uncontrolled artefacts while recording can be observed in the absence of PEDOT:PSS, that is, 1*vs*2 20% HEA‐co‐PEGDA/polyDES. In order to confirm that the signal amplitude acquired in 1*vs*2 20%HEA‐co‐PEGDA/polyDES was due to artifacts, the variability of the signal during the muscle contraction was evaluated (Figure 6D; Figures S13, Supporting Information). As observed in Figure 6D, the envelope variability voltage acquired from the histogram of magnitude values during muscle contraction displays clearly more polydispersed signal when 20% PEGDA‐co‐HEA/polyDES electrodes are used for recording while a more controlled and sharp envelope when PEDOT:PSS is in contact with the skin. Hence, we can confirm that the presence of PEDOT:PSS as a mixed conductor in combination with a polyDES as an ionic conductor minimizes the skin impedance and remarkably improves biosignal acquisition. Finally, polyDES has also been demonstrated to improve electrode stability in dry conditions. As we have pointed out, PEDOT:PSS/20% HEA‐co‐PEGDA is a more stable copolymer than the homopolymer (PEDOT:PSS/100% PEGDA); however, after ≈24 h, it gets completely dry, the structure tends to collapse, creating holes and imperfections (see Figure S16, Supporting Information). When the polyDES coated the conducting protuberances, the electrode demonstrated activity after 2 months of its fabrication, addressing long‐term stability and retaining the same SNR values. The electrodes remained exposed to air conditions during this period. The SNR of the electrodes after 90 days remained practically equal to 1 day of age. (see Figure 6E; Figure S17, Supporting Information), with 3.44 ± 0.1, 7.22 ± 0.6, and 3.09 ± 0.2 for 5, 10, and 20 kg, respectively, confirming its excellent performance over time.

We also demonstrated the electrode's ability to record electrocardiography (ECG), acquiring waveforms that accurately reflect the P, QRS, and T curves. (see Figure S18, Supporting Information).

## 4 Conclusion

Overall, in this article, we addressed some of the major limitations of light‐based 3D printing. First, we demonstrated a new method for multi‐material printing in one‐go with a commercially available 3D printer. The approach relies on the modification of the Z‐value and the iterative change of the material inside the VAT. Secondly, the optimization of a functional ink having conducting PEDOT:PSS printing was shown, along with its combination with multi‐material printing. The multi‐material printing overcomes the mechanical stability of PEDOT:PSS structures, allowing the manufacturing of miniaturized bio‐shaped topographies with high resolution (≈100 µm). Mushroom‐shaped protuberances were combined with functional additive polyDES and optimized to confer simultaneously adhesivity and ionic conductivity. Finally, bio‐shaped multi‐material printing devices of optimum height, 1*vs*2 PEDOT:PSS/PEGDA‐co‐HA/polyDES were manufactured to record EMG signals on the forearm. The performance of the device was compared with a non‐electronic conductor device, deciphering the importance of mixed conduction. The electrodes were stable 90 days after manufacturing without losing their performance. The engineering of conducting inks, smart additives, and this manufacturing process offers a vast potential for future larger‐area wearable electrodes, prosthetics, or implantable applications with multi‐factorial properties. We envision our methodology as a universal approach to manufacturing 3D structures without the need to generate a new printer or the necessity of deep knowledge in electronics, software engineering, or mechanical engineering. We strongly believe this is an exciting step toward wearable electronics and 3D printing, with plenty of room for improvement and modifications.

## Conflict of Interest

The authors declare no conflict of interest.

## Supporting information

Supporting Information

## Data Availability

The data that support the findings of this study are available from the corresponding author upon reasonable request.
