# Supplementary material for: Light‐Based 3D Multi‐Material Printing of Micro‐Structured Bio‐Shaped, Conducting and Dry Adhesive Electrodes for Bioelectronics
Source: Adv Sci (Weinh). 2024 Jan 22;11(27):2306424. doi: 10.1002/advs.202306424 (PMC11251555; doi:10.1002/advs.202306424)
Supplement: Supplementary file 1 — Supporting Information [file ADVS-11-2306424-s001.pdf]

ADVANCED SCIENCE
Open Access

Supporting Information

for *Adv. Sci.*, DOI 10.1002/advs.202306424

Light-Based 3D Multi-Material Printing of Micro-Structured Bio-Shaped, Conducting and Dry Adhesive Electrodes for Bioelectronics

Antonio Dominguez-Alfaro, Eleni Mitoudi-Vagourdi, Ivan Dimov, Matias L. Picchio, Naroa Lopez-Larrea, Jon Lopez de Lacalle, Xudong Tao, Ruben Ruiz-Mateos Serrano, Antonela Gallastegui, Nikolaos Vassardanis, David Mecerreyes and George G. Malliaras**

Supporting Information

Light-Based 3D Multi-Material Printing of Micro-Structured Bio-Shaped, Conducting and Dry Adhesive Electrodes for Bioelectronics

Antonio Dominguez-Alfaro,[a,b] Eleni Mitoudi Vagourdi,[a] Ivan Dimov,[a] Matías L. Picchio,[b] Naroa Lopez-Larrea,[b] Jon Lopez de Lacalle,[b] Xudong Tao,[a] Ruben Ruiz-Mateos Serrano,[a] Antonela Gallastegui,[b] Nikolaos Vassardanis,[d] David Mecerreyes[b,c] and George G. Malliaras*[*,a]*

[a] Electrical Engineering Division, Department of Engineering, University of Cambridge, 9 JJ Thomson Ave, Cambridge, CB3 0FA, UK

[b] POLYMAT, University of the Basque Country UPV/EHU, Avenida Tolosa 72, Donostia-San Sebastián, Gipuzkoa 20018, Spain

[c] IKERBASQUE, Basque Foundation for Science, 48009, Bilbao, Spain

[d] VASSARDANIS L.P. , Acharnon 17, 14561, Kifisia, Greece

Email: ad2151@cam.ac.uk and gm603@cam.ac.uk

KEYWORDS: PEDOT:PSS, DLP 3D PRINTING, MULTI-MATERIAL PRINTING, ADHESION, BIOELECTRONICS

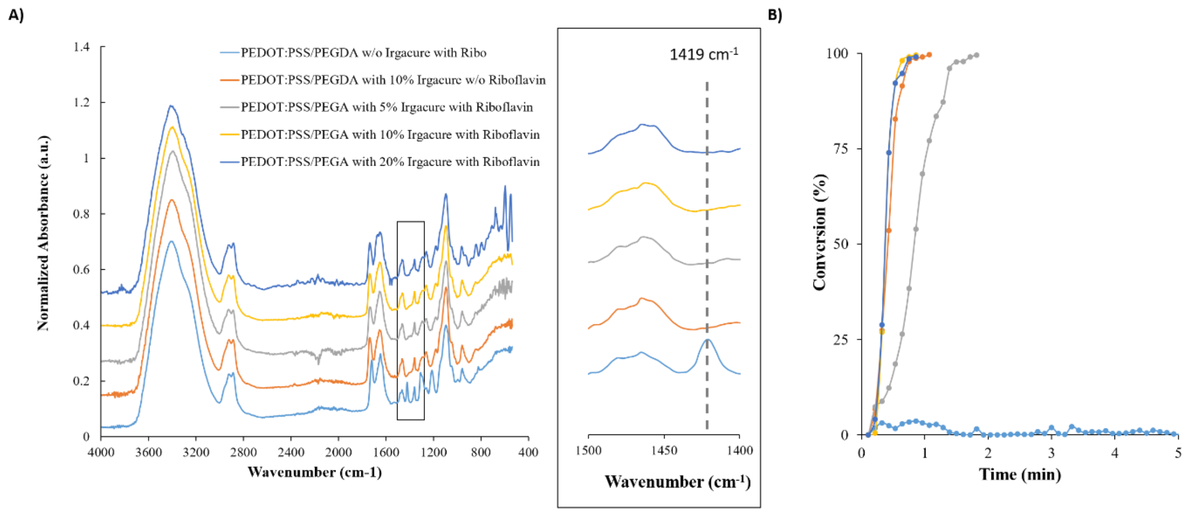

Figure S1. A) ATR-FTIR spectra of PEDOT:PSS/PEGDA without and with different amounts of riboflavin: Irgacure 2289 (1:1 %v/v) as sensitizer:photoinitiator. The riboflavin as a sensitizer is also studied. In the inset, the peak of the C=C after polymerization can be observed depending on the polymerization conditions. B) Conversion kinetics of the same inks studied in the C=C range at different times during the photopolymerization process.

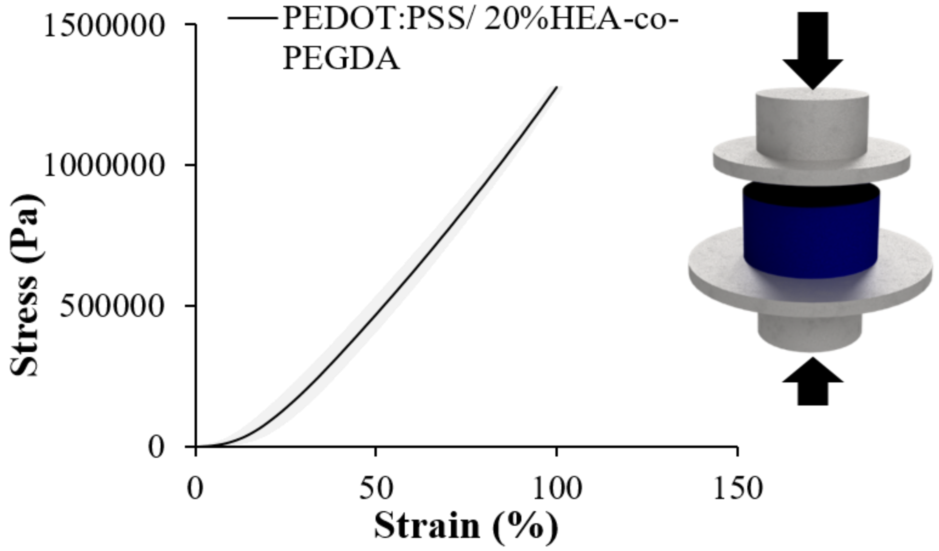

Figure S2. Stress-strain curve of PEDOT:PSS/ 20% HEA-co-PEGDA obtained for compression test after swelling.

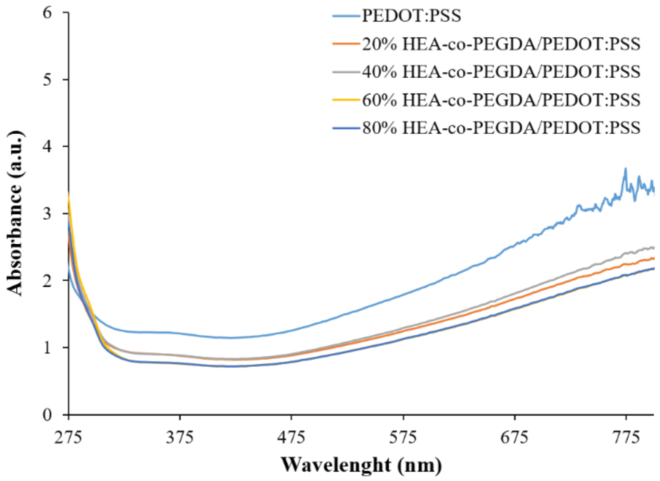

Figure S3. UV-vis spectra of different PEDOT:PSS/Acrylate films of 50 µm of thickness, compared to 50 v/v% PEDOT:PSS solution.

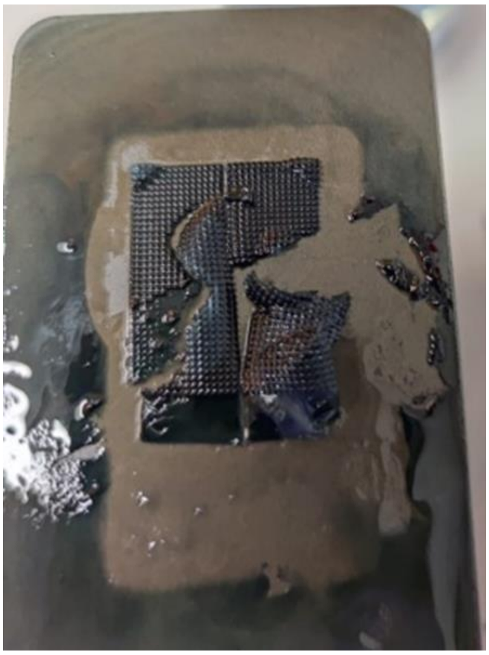

Figure S4. Photo of PEDOT:PSS/PEGDA printed on top of the electrode, demonstrating its poor mechanical properties when printed directly on the holder.

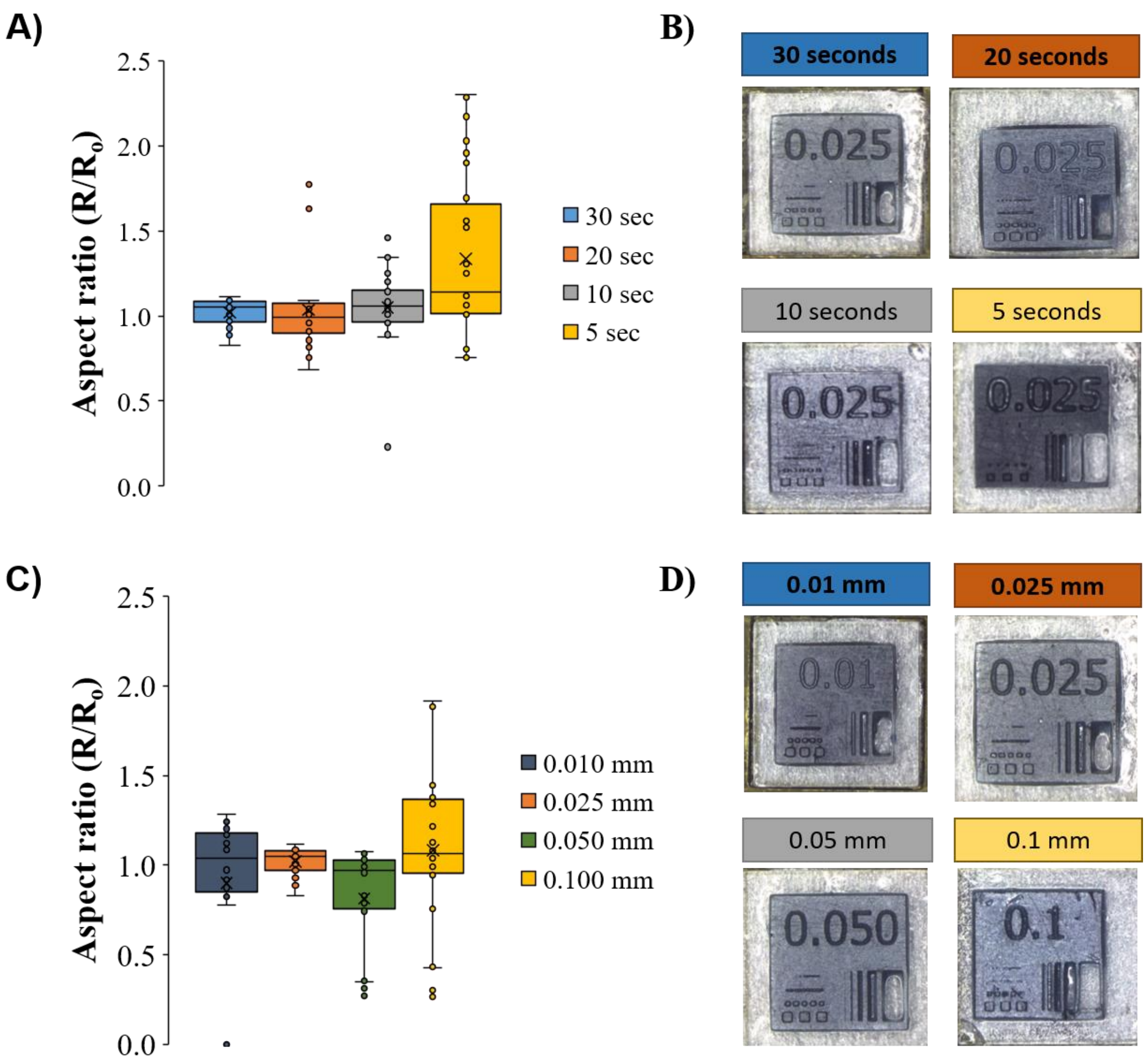

Figure S5. A) Shape fidelity measured by Aspect ratios (R/R_o) for 25 µm layer height at different exposure times *i.e.* 5, 10, 20, 30. B) Photo of the resulted patterns, showing time-dependent resolution. C) Shape fidelity measured by Aspect ratios (R/R_o) for different layer height *i.e.* 0.01, 0.025, 0.050 and 0.1 at same exposure time of 30 s. D) Photo of the resulted patterns, showing layer height-dependent resolution. 3D patterns were designed with Autodesk Inventor (Square Base = 6.0 mm side × 5.71 mm thickness) (Note: R is the experimental size of features measured under an optical microscope and R_o is the real size of same features as designed in Autodesk Inventor)

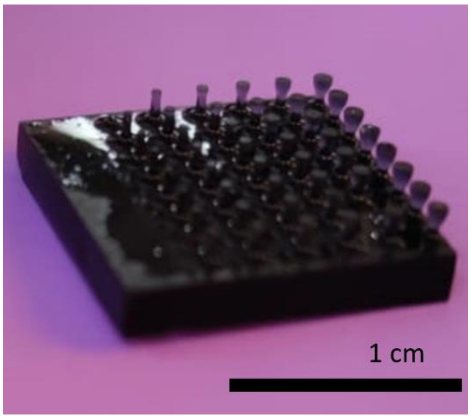

Figure S6. A) 3D printed PEDOT:PSS/20% HEA-co-PEGDA with different angle-test topographies.

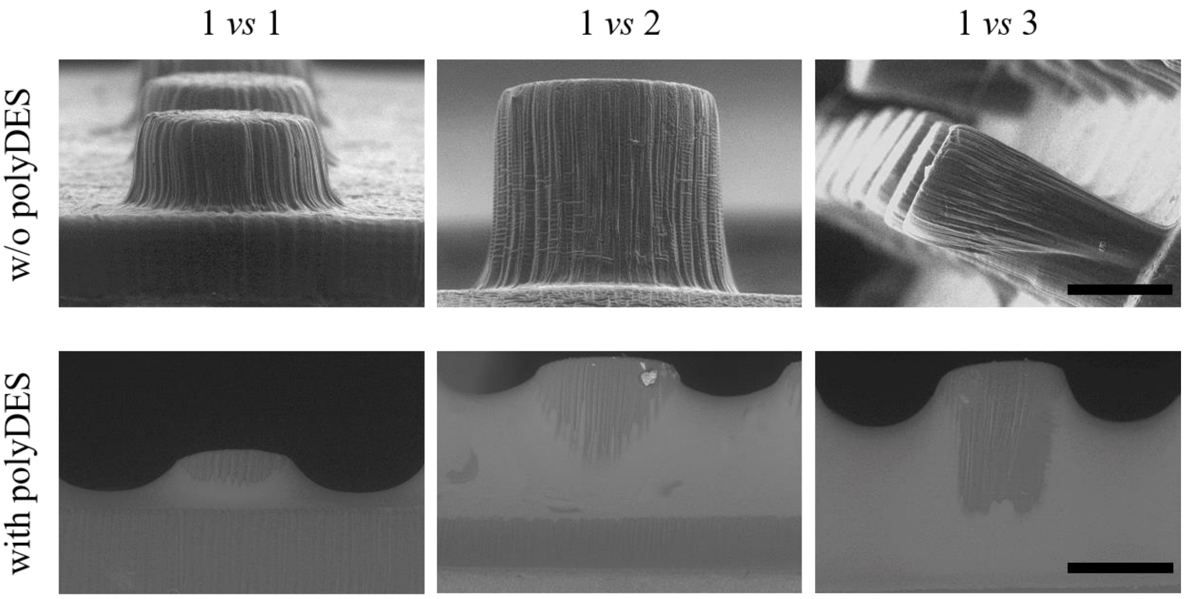

Figure S7. SEM images comparing different protuberances height with and without polyDES (Scale bar: 300 µm).

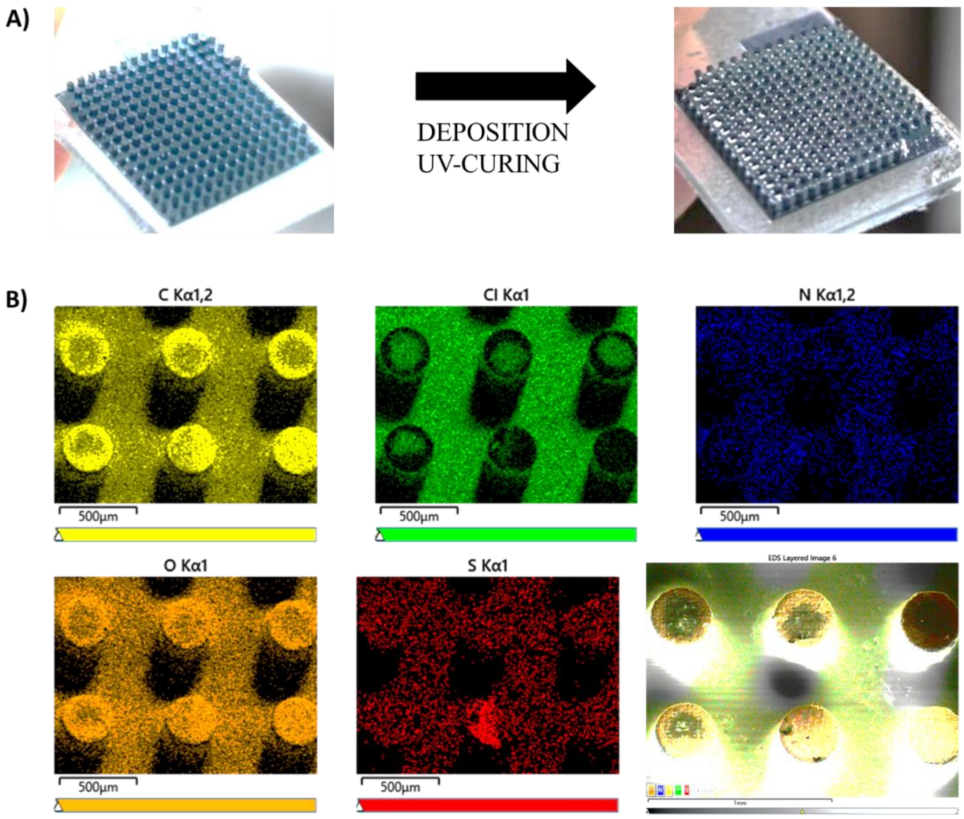

Figure S8. A) PEDOT:PSS/20% HEA-co-PEGDA mushroom electrode appearance pre- and after-polyDES deposition and curing and B) SEM-EDX of the same electrodes where carbon, chlorine, nitrogen, oxygen, surlphur and all the elements merged

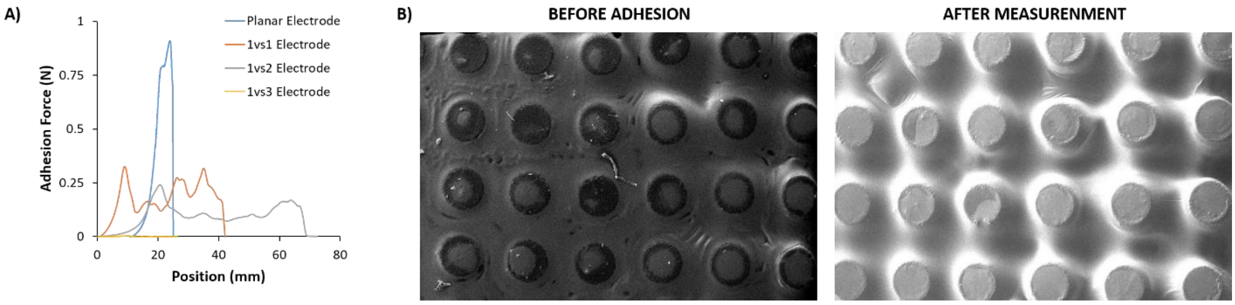

Figure S9. A) Comparison of shear-lap measurements of PEDOT:PSS/20% HEA-co-PEGDA/polyDES for planar, 1vs1, 1vs2 and 1vs2 Mushroom's electrodes. B) SEM Front view of 1*vs*2 PEDOT:PSS/20% HEA-co-PEGDA/polyDES before and after the adhesion measurements.

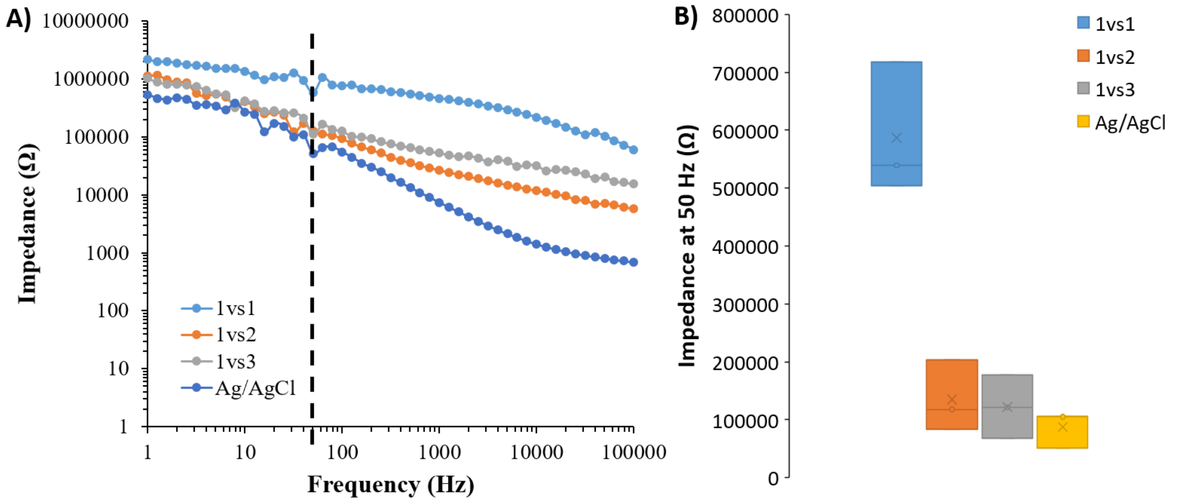

Figure S10. A) EIS comparison on skin of PEDOT:PSS/ 20% HEA-co-PEGDA/polyDES mushroom electrodes with 1vs1, 1vs2 and 1vs3 configurations and Ag/AgCl measured on the skin. B) Box-plot of impedance of same specimens at 50 Hz.

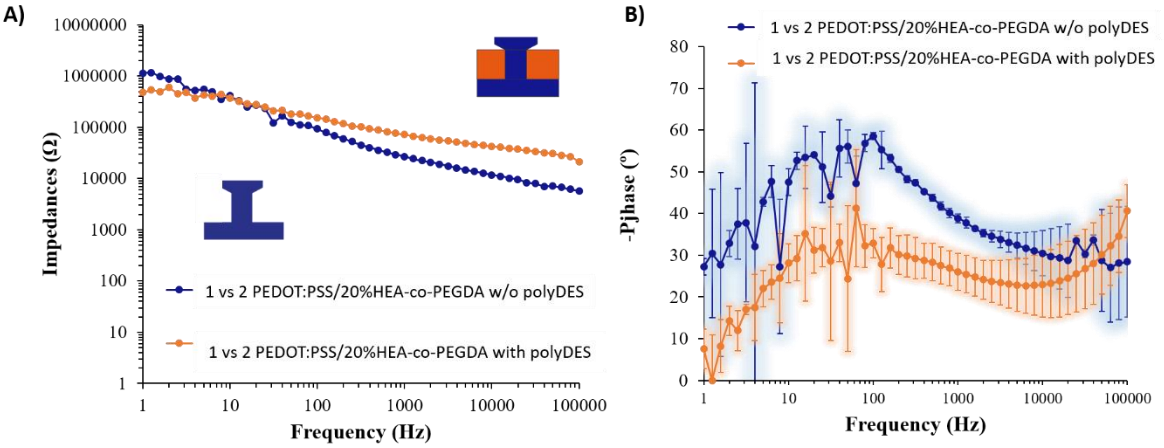

Figure S11. A) EIS and B) –Phase(°) comparison of 1vs2 Mushroom electrodes measured on the skin with and without the polymerized DES.

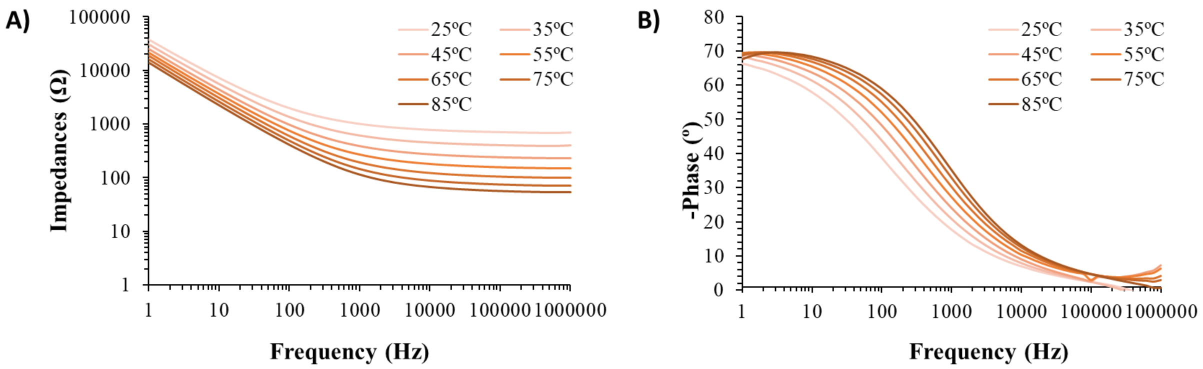

Figure S12. A) Impedances and B) –Phase(°) comparison of polyDES at different temperatures in a sandwich conformation cell.

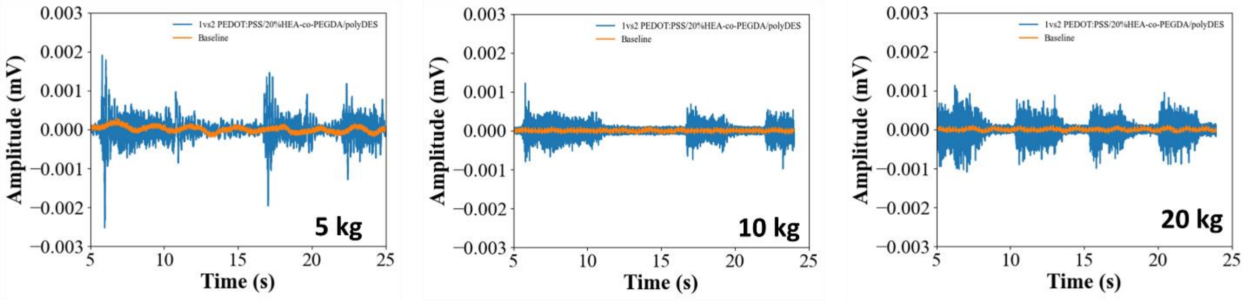

Figure S13. Twenty five seconds of EMG recordings on the forearm of 1vs2 PEDOT:PSS/20%HEA-co-PEGDA/polyDES electrodes when 5, 10 and 20 kg of grip were applied.

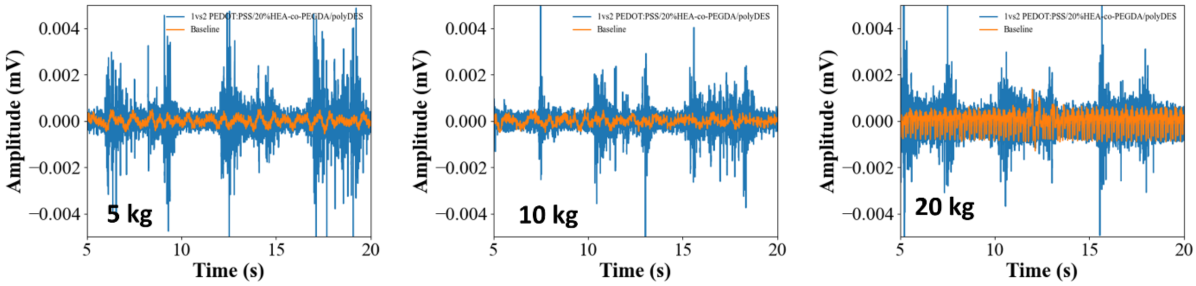

Figure S14. Twenty seconds of EMG recordings on the forearm of 1vs2 20%HEA-co-PEGDA/polyDES electrodes when 5, 10 and 20 kg of grip were applied.

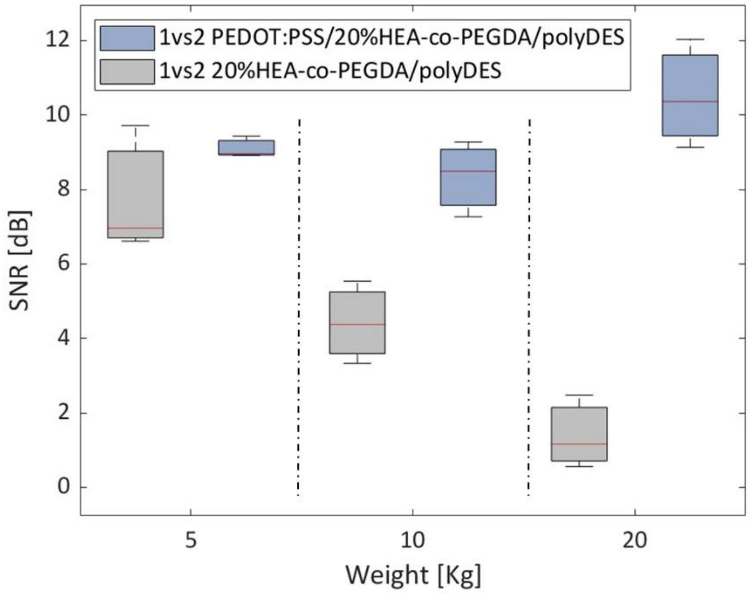

Figure S15. Twenty seconds of EMG recordings on the forearm of 1vs2 20%HEA-co-PEGDA/polyDES electrodes when 5, 10 and 20 kg of grip were applied.

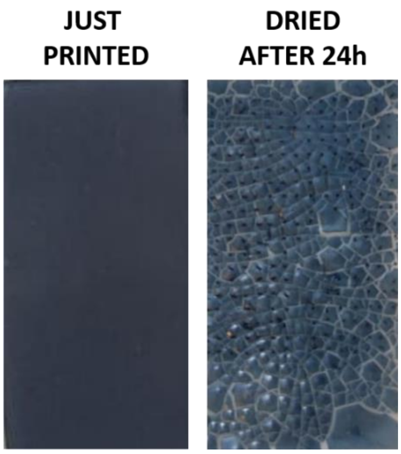

Figure S16. Photos of planar electrodes made of PEDOT:PSS/HEA-co-PEGDA without the polyDES, just printed and after 24h dried at room temperature.

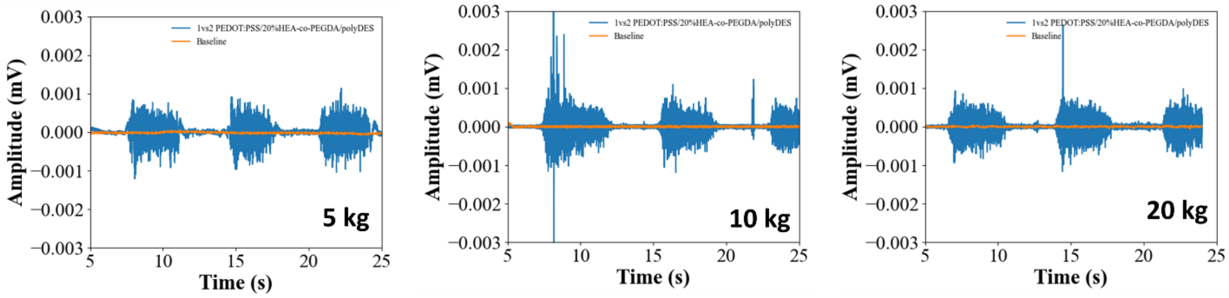

Figure S17. 25 seconds of EMG recordings on the forearm of 1vs2 PEDOT:PSS/20%HEA-co-PEGDA/polyDES electrodes when 5, 10 and 20 kg of grip were applied 90 days after the date of printing.

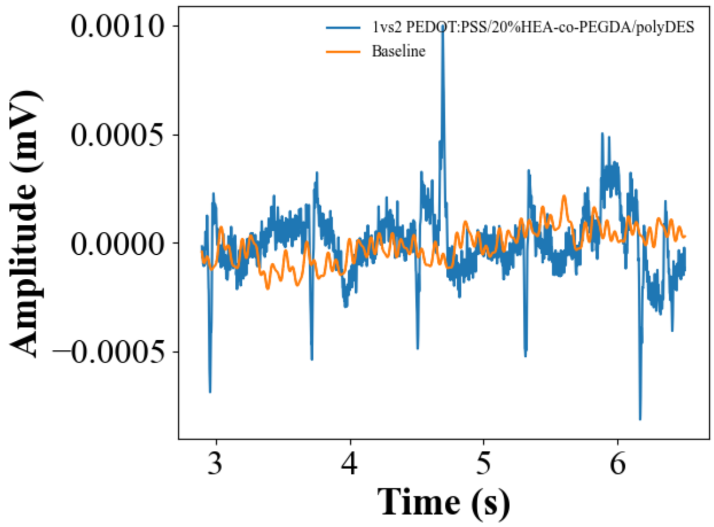

Figure S18. ECG recordings on the chest of 1vs2 PEDOT:PSS/20%HEA-co-PEGDA/polyDES electrodes.

	Type	Name of multi-material approache	Requirements and limitations	Materials printed	Minimum resolution shown
[1]	Extrusion-based printing	Voxelated printing	-3D printed print heads<br>-Pneumatic controllers<br>-Suitable inks with apparent viscosity 100-1000 Pa·s)	Silicone, wax and epoxy inks.	250-320 μm
[2]		Multi-nozzle adaptive	- 3D printed print heads<br>- Pneumatic and motor controllers<br>- Additional profilometers, and/or blue light source<br>Suitable inks with apparent viscosity 3–5 Pa·s)	Triblock copolymer (Pluronic F127), urethane dimethacrylate, isodecyl acrylate and hydroxyethyl methacrylate	>840 μm
[3]	Extrusion- and light-based printing combined	Rotational printing	-3D printed shell–fan-core print heads<br>-Pneumatic, UV-lamp and motor controllers<br>-Additional UV-curing for the acrylate-polymerization<br>-Suitable inks with apparent viscosity $1 \cdot 10^4$ Pa·s)	PDMS and 1,6-Hexanediol diacrylate/ carbon black	>800 μm
[4]	Light-based printing	Injection continuous liquid interface (iCLIP)	-All-printer controllers and elements (motor, UV, micro mirror device, diode, chips…)<br>-Coordinated with Arduino, besides software for graphical user interface<br>-Viscosities range from 0.1 to 7 Pa·s<br>-Limited printing area (>700 mm^2)	Isobornyl methacrylate, bisphenol A ethoxylated acrylate, multiwalled carbon nanotubes and bisphenol A glycidyl methacrylate	250-1500 μm

[5]		DLP-based multi-material printer with air-jetting to remove non-reacted monomer	-All-printer controllers and elements (motor, holder, UV, micro mirror device, diode, chips…)<br>-Air-injection to remove residues	Acrylamide, PEGDA, tango elastomer®, vero rigid polymer®, and Agilus®	>100 µm
[6]		DLP-based multi-material printer with centrifugation to remove non-reacted monomer	-All printer controllers and elements (motor, holder, UV, micro mirror device, diode, chips…)<br>-Centrifugation to remove residues	Vero resin and ABS plus from Stratasys®Ltd, acrylamide, PEGDA, tert-butyl acrylate and ceramic composite	<100 µm
[7]		Multi-material 3D laser micro-printing with a integrated microfluidic system	- Direct laser writing system<br>-Microfluidic chamber holder<br>-Air-jetted system, photoresist injectors, pumping system	Pentaerythritol tetraacrylate (PETA), TDDDA,	<5 µm
		Our work	- Commercially available 3D printer with Z-modulation option	PEGDA, PEDOT:PSS and HEA	<100 µm

Table S1. Comparative of all the multi-material printing approaches shown in the introduction section and its comparative with this project

Bibliography

[1] M. A. Skylar-Scott, J. Mueller, C. W. Visser, J. A. Lewis, *Nature* **2019**, *575*, 330.

[2] S. G. M. Uzel, R. D. Weeks, M. Eriksson, D. Kokkinis, J. A. Lewis, *Adv. Mater. Technol.* **2022**, *7*, 2101710.

[3] N. M. Larson, J. Mueller, A. Chortos, Z. S. Davidson, D. R. Clarke, J. A. Lewis, *Nature*

2023, *613*, 682.

[4] G. Lipkowitz, T. Samuelsen, K. Hsiao, B. Lee, M. T. Dulay, I. Coates, H. Lin, W. Pan, G. Toth, L. Tate, E. S. G. Shaqfeh, J. M. DeSimone, *Sci. Adv.* **2023**, *8*, eabq3917.

[5] Q. Ge, Z. Chen, J. Cheng, B. Zhang, Y.-F. Zhang, H. Li, X. He, C. Yuan, J. Liu, S. Magdassi, S. Qu, *Sci. Adv.* **2021**, *7*, eaba4261.

[6] J. Cheng, R. Wang, Z. Sun, Q. Liu, X. He, H. Li, H. Ye, X. Yang, X. Wei, Z. Li, B. Jian, W. Deng, Q. Ge, *Nat. Commun.* **2022**, *13*, 7931.

[7] F. Mayer, S. Richter, J. Westhauser, E. Blasco, C. Barner-Kowollik, M. Wegener, *Sci. Adv.* **2023**, *5*, eaau9160.
